# Using a Mobile App for Monitoring Post-Operative Quality of Recovery of Patients at Home: A Feasibility Study

**DOI:** 10.2196/mhealth.3929

**Published:** 2015-02-12

**Authors:** John L Semple, Sarah Sharpe, M Lucas Murnaghan, John Theodoropoulos, Kelly A Metcalfe

**Affiliations:** ^1^Women\'s College HospitalDepartment of SurgeryUniversity of TorontoToronto, ONCanada; ^2^Institute of Health PolicyManagement and EvaluationUniversity of TorontoToronto, ONCanada; ^3^The Hospital for Sick ChildrenDivision of OrthopedicsToronto, ONCanada; ^4^University of TorontoDepartment of SurgeryToronto, ONCanada; ^5^Women's College Research InstituteToronto, ONCanada; ^6^University of TorontoLawrence S. Bloomberg Faculty of NursingToronto, ONCanada

**Keywords:** outpatient, recovery, care, post-operative, smartphone, technology, mobile

## Abstract

**Background:**

Mobile apps are being viewed as a new solution for post-operative monitoring of surgical patients. Mobile phone monitoring of patients in the post-operative period can allow expedited discharge and may allow early detection of complications.

**Objective:**

The objective of the current study was to assess the feasibility of using a mobile app for the monitoring of post-operative quality of recovery at home following surgery in an ambulatory setting.

**Methods:**

We enrolled 65 consecutive patients (n=33, breast reconstruction surgery; n=32, orthopedic surgery) and asked them to use a mobile phone daily to complete a validated quality of recovery scale (QoR-9) and take photographs of the surgical site for the first 30 days post-op. Surgeons were asked to review patient-entered data on each patient in their roster daily. A semistructured questionnaire was administered to patients and surgeons to assess satisfaction and feasibility of the mobile device.

**Results:**

All 65 patients completed the study. The mean number of logins was 23.9 (range 7-30) for the breast patients and 19.3 (range 5-30) for the orthopedic patients. The mean number of logins was higher in the first 14 days compared to the 15-30 days post-op for both breast patients (13.4 vs 10.5; *P*<.001) and for the orthopedic patients (13.4 vs 6.0; *P*<.001). The mean score for overall satisfaction with using the mobile device was 3.9 for breast patients and 3.7 for orthopedic patients (scored from 1 (poor) to 4 (excellent)). Surgeons reported on the easy-to-navigate design, the portability to monitor patients outside of hospital, and the ability of the technology to improve time efficiency.

**Conclusions:**

The use of mobile apps for monitoring the quality of recovery in post-operative patients at home was feasible and acceptable to patients and surgeons in the current study. Future large scale studies in varying patient populations are required.

## Introduction

There is a growing body of evidence that supports the use of mobile apps in health care interventions [1]. These include the use of apps for smoking cessation [2], other behavior change programs such as exercise and weight management [3,4], and self-management of long-term conditions such as diabetes [5]. Text messaging is being used to provide health education [6], to issue reminders for appointments [7], and to improve the efficiency of health systems overall [8,9]. However, there is relatively little research on the feasibility or effectiveness of downloadable apps or software for mobile phones (specifically smartphones) for the remote monitoring of patients following surgery [10]. Surgical populations differ from previous groups monitored with mobile phone technology but are highly appropriate in that they have a homogenous set of standard indicators of what constitutes quality of recovery, common touch points regarding care professionals and hospitals, and have a limited recovery period [11].

Modern ambulatory surgical units are performing more complex surgical procedures due to new approaches in pain control, the introduction of techniques that reduce the peri-operative stress response, and the use of minimally invasive surgical techniques [11].

The first 30 days following surgery have been identified as a major focus area in health care [12,13]. The majority of post-operative complications develop during this time period [13]. In addition, during this 30-day post-op period, many of the unexpected visits to the emergency department and re-admissions to hospital occur. These unexpected visits and re-admissions to hospital cost the health care systems in North America billions of dollars annually [13]. Modern surgical initiatives such as expedited discharge and fast-tracking programs require appropriate methodology to monitor and maintain quality of recovery while the patients recover at home [14,15].

Mobile phone monitoring of patients in the post-operative period may not only allow faster discharge of patients from hospital but also shed insight into the patient’s experience while at home and provide a mechanism for the early detection of developing complications. In addition, there would be numerous benefits for surgeons and care providers in having the ability to monitor patients remotely as well as potential time saved in replacing face-to-face visits with virtual visits.

We performed a feasibility study to determine if mobile apps can be used to monitor a patient’s recovery at home during the immediate post-operative period.

##  Methods

### Overview

This prospective cohort study was conducted with ambulatory care patients undergoing breast reconstruction (breast) or orthopedic arthroscopic anterior cruciate ligament repair (ACL) surgery. The pilot study began in October 2011. All patients registered for breast reconstruction and orthopedic surgery were screened for inclusion in the study until the desired number of participants was obtained (minimum 60 patients—30 breast reconstruction and 30 orthopedic surgery). Patients were selected based on three inclusion criteria: (1) ages 18-75, (2) non-smoker status (in breast reconstruction patients as there is a high complication rate in smokers), and (3) ability to communicate in English. Patients were excluded from the study if they suffered from chronic pain or psychiatric disturbances, took narcotic (morphine-like) medication for pain on a regular basis, or had an allergy to local anesthetics or morphine-like medications. All patients received an information sheet discussing the purpose of the study and had the opportunity to discuss the process with the study coordinator. After written consent was obtained by the study coordinator, each patient met with the coordinator for approximately 30-45 minutes before their surgery to learn how to use the mobile device and review the different indicators they would be asked to answer on the mobile device each day. Patients were also shown how to take a picture of their surgical wound site using the mobile device.

Prior to discharge, patients were given either a smartphone or a tablet with half of each study arm receiving one or the other. Software was provided by QoC Health Inc (Toronto), devices were provided by Samsung, and network time was provided by Rogers Communications. Patients practiced taking photos with the study coordinator to ensure they understood how to take pictures with the device. In addition to the one-on-one consultation with the study coordinator, each participant was provided with an education booklet with details on how to use the mobile app device and answer questions regarding security and confidentiality. The education booklet also included illustrations on how to frame the body and angle the camera to take pictures. These parameters were based on the most effective view required by surgeons to assess the wound site. Study participants continued to have their regularly scheduled face-to-face post-operative visits with the surgeon in keeping with normal care processes.

The three participating surgeons used a mobile interface or desktop computer to access patient data. Scores on question items that fell outside the normal range of defined parameters were immediately flagged in the database for quick viewing. Being flagged meant that the app sensed an extreme on the value scale as a response. The app would alert the surgeon by sending a “flag” to the surgeon (care provider). In addition, the app would report the list of patients on the roster so that the “flagged” patient would be at the top of the list and be highlighted in red. The surgeon could then phone the patient to enquire why the extreme score had been registered. The app updated every 5 minutes. Results and flags were reviewed, and if needed, patients were contacted by the surgeon and/or nurse. Daily photographs were reviewed by surgeons to assess the recovery of the surgical site and wound and to determine if healing was progressing normally and without complications.

Four sets of data were gathered for this study including (1) data from the mobile app that was entered by patients in the pilot study while recovering at home, (2) post-recovery follow-up feedback surveys with patients, (3) post-recovery interviews with patients, and (4) post-pilot feedback surveys with participating surgeons.

###  Mobile App Data

The mobile app recovery indicators included a visual analogue scale (VAS) for pain and Likert questions from the Quality of Recovery (QoR-9) questionnaire. The QoR-9 is an earlier version of the Quality of Recovery (QoR-40) questionnaire, which has been extensively utilized and validated to assess the quality of life of patients after surgery [14]. The QoR-40 is composed of 40 items organized into six dimensions: emotional state, physical comfort, psychological support, physical independence, pain, and a global score [14]. Despite the comprehensiveness of the QoR-40, the feasibility of using this questionnaire as a daily self-administered assessment tool is limited due to survey length. In consideration of time and the frequency of conducting the assessments, the QoR-9 is much more appropriate for the purpose of daily remote monitoring and provides insight into patient quality of recovery and outcomes of care. In this study, all nine questions from the QoR-9 served as indicators; however, one item (“been able to pass urine and no trouble with bowel movements”) was split into two question items addressing urine function and bowel function separately. Reporting began from the day of discharge up to a period of 6 weeks dependent on patient recovery time for each procedure. The patients were asked to complete the app survey once per day at the outset but not reminded in any way. At the outset, we asked the patients to answer the survey in the morning when they took their first set of pain medications. This was in an attempt to standardize the responses.

**Figure 1:**
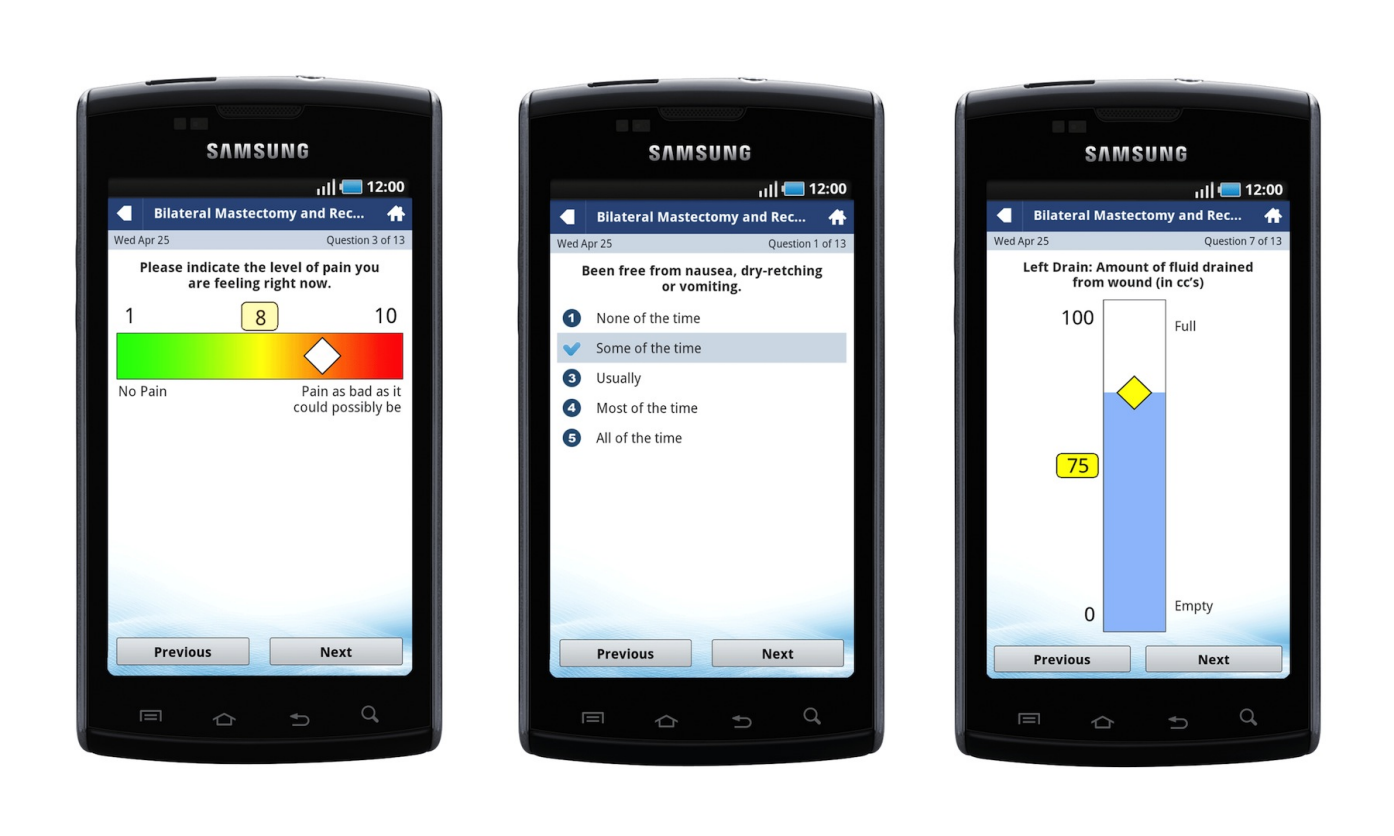
Examples of the touch screen interface of the patient portal, including the visual analogue pain scale, an example of the QoR 9 question on postoperative nausea, and the visual analogue scale for fluid in the postoperative surgical drains.

Table 1 lists the indicators on the mobiles devices for breast reconstruction and orthopedic patients in the pilot, in addition to the scores that elicited a “flag” for the surgeons. Being flagged meant that the app sensed an extreme on the value scale as a response. The app would alert the surgeon by sending a “flag” to the surgeon (care provider). In addition, the app would list the patients on the roster so that the “flagged” patient would be at the top of the list and be highlighted in red. The surgeon could then phone the patient to enquire why the extreme score had been registered. The app updated every 5 minutes. Figure 1 depicts the screenshots and provides an example of the indicators as viewed on the mobile devices.

**Table 1:** List of indicators on the mobile device.

Type	Question	Scale	Flag
Anxiety	How anxious (worried, nervous) do you feel?	1-not at all anxious, 2-a little anxious, 3-moderately anxious, 4-very anxious, 5-extremely anxious	4 & 5
Pain	Please indicate the level of pain you are feeling right now.	Visual Analogue Scale 1-10	5-10
Drain (breast)	Amount of fluid drained from wound (in cc’s)	1-100 cc’s sliding scale (one drain per breast and side of abdomen)	50-100
QoR	Had a feeling of general well being	1-all of the time, 2-most of the time, 3-usually, 4-some of the time, 5-none of the time	4 & 5
QoR	Had support from others	1-all of the time, 2-most of the time, 3-usually, 4-some of the time, 5-none of the time	4 & 5
QoR	Been able to understand instructions and advice. Not being confused	1-all of the time, 2-most of the time, 3-usually, 4-some of the time, 5-none of the time	4 & 5
QoR	Been able to look after personal toilet and hygiene unaided	1-all of the time, 2-most of the time, 3-usually, 4-some of the time, 5-none of the time	4 & 5
QoR	Been able to pass urine	1-all of the time, 2-most of the time, 3-usually, 4-some of the time, 5-none of the time	4 & 5
QoR	Had normal bowel function	1-all of the time, 2-most of the time, 3-usually, 4-some of the time, 5-none of the time	4 & 5
QoR	Been able to breathe easily	1-all of the time, 2-most of the time, 3-usually, 4-some of the time, 5-none of the time	4 & 5
QoR	Been free from headache, backache or muscle pains	1-all of the time, 2-most of the time, 3-usually, 4-some of the time, 5-none of the time	4 & 5
QoR	Been free from nausea, dry-retching, or vomiting	1-all of the time, 2-most of the time, 3-usually, 4-some of the time, 5-none of the time	4 & 5
QoR	Been free from experiencing severe pain or constant moderate pain	1-all of the time, 2-most of the time, 3-usually, 4-some of the time, 5-none of the time	4 & 5
Mobility (ortho)	How difficult is it to stand on your leg?	1-not at all difficult, 2-slightly difficult, 3-moderately difficult, 4-very difficult, 5-extremely difficult	4 & 5
Mobility (ortho)	How difficult is it to walk on your leg?	1-not at all difficult, 2-slightly difficult, 3-moderately difficult, 4-very difficult, 5-extremely difficult	4 & 5
Mobility (ortho)	How difficult is it to go up and down stairs?	1-not at all difficult, 2-slightly difficult, 3-moderately difficult, 4-very difficult, 5-extremely difficult	4 & 5
Picture	Take a photograph of your procedure site. You can add several photos.	N/A	N/A

### Post-Recovery Follow-Up Survey for Patients

During the second follow-up visit to the surgeon, the mobile device was returned and patients were invited to fill in a post-recovery survey evaluating their recovery as well as their experience using the mobile device. The survey contained 33 questions including the same nine recovery indicators as the mobile app as well as questions pertaining to anxiety, desire to contact health care professionals, satisfaction with quality of care, satisfaction with the mobile device, and the patients’ willingness to pay for the app in the future. The study coordinator was available while patients filled in the survey to provide clarification when required and answer patient questions about the study. In the event the patient was unable to complete the survey without assistance, the survey was read aloud by the coordinator and completed in a structured interview manner.

###  Post-Recovery Interview With Patients

Patients in the study were also invited to participate in a study evaluation interview. The interview focused on the patient recovery experience using the mobile device, user-friendliness of the mobile device and app, and suggestions for improvement.

###  Post-Pilot Surveys With Participating Surgeons

After the study was complete, the 3 participating surgeons were asked to fill out a post-pilot survey with 13 open-ended questions evaluating their post-operative recovery experience using the QoC Health technology platform. This survey included questions on overall experience, user-friendliness of the portal, changes that could improve the app and portal, potential impact on care and reduction of patient post-op visits, and potential for the solution to identify complications.

###  Ethical Considerations

Women’s College Hospital Research Ethics Board approval for the pilot study was granted in July 2011. As there was no change in the current medical standard of care, there was no risk to patients by participating in the study. No patient was discharged from the hospital unless they met the standard discharge criteria applied to all patients, and all standard practices of post-surgical care were followed.

### Patient Confidentiality

To ensure patient confidentiality, all hard copies of the data (ie, surveys and interview notes) were stored in locked filing cabinets in the investigator’s office at Women’s College Hospital with access restricted. All patients were coded, so that identifiers were absent from survey and interview data. Data sheets containing subject identifiers as well as subject identifications numbers were stored separately from data sheets containing subject identification numbers only. No identifiers were or will be included on any hard copy data sheets that link with health information (ie, only identification numbers are used), and password-protected databases are used.

Pictures of patients were related to the surgical site only, and no patient identifiers were used. By using managed devices (patients were given a mobile phone), a “locked down” subscriber identity module (SIM) card was used. The photographs were kept on a secure internal file folder and not available for viewing in the “Gallery” aspect of the mobile phone apps.

Health Canada was consulted as to whether this monitoring concept on a mobile phone would be considered a classification of “medical device”. Because it is not diagnostic and is essentially “monitoring stored forward” data, it is not considered a “device”.

Canadian Medical Protective Agency was also consulted in regards to medical legal liability and the participating surgeons. Their only stipulation was that of maintaining standards of privacy and security of patient data.

### Data Security on a Mobile Platform

Patient data collected using the mobile app is double encrypted on the server and the phone. Designed from the “ground up” to ensure security and privacy, the app conforms to leading health care audit and interoperability standards including the Personal Health Protection Act, Health Level Seven International (HL7), Information Technology Infrastructure Library (ITIL), and Statement on Auditing Standards No. 70 (SAS70). Multiple layers of encryption, including resting state advanced encryption standard (AES) encryption, in transmission content encryption using unique per patient public/private key pairs, and in transmission transport layer security/secure sockets layer (TLS/SSL) protocol encryption were applied to maintain the highest level of patient confidentiality as possible. Modern infrastructure design leveraging distributed infrastructure as a service (IaaS) and cloud computing services (SaaS) for seamless accessibility, redundancy, and scalability were also utilized.

###  Analysis

Both quantitative and qualitative analyses were performed. Descriptive statistics were generated for demographic variables. Overall satisfaction with use of mobile device was scored from 1 (poor) to 4 (excellent), and means were generated. Frequencies were generated for categorical variables. Qualitative information from the evaluation survey and interviews were assessed for common themes.

##  Results

###  Demographics

A total of 38 breast patients were approached for participation, and 33 (87%) consented to participate. A total of 40 orthopedic patients seeking arthroscopic anterior cruciate ligament reconstruction were approached, and 32 (80%) consented to participate. Reasons for not participating included ineligibility (n=2), surgery cancellations (n=3), inability to provide a device due to timing of surgery (n=3), and patient preference not to participate (n=5). We enrolled 65 patients in the study, and Table 2 shows a summary of the demographic data for both orthopedic and breast patients. All of the patients completed the study protocol.

**Table 2:** Patient demographic information.

Characteristics			n (%)
**Breast reconstruction (n=33)**			
	**Gender**		
		Male	0 (0)
		Female	33 (100)
	**Age in years**		
		Mean age	48
		Age range	32-68
**Orthopedic-arthroscopic anterior cruciate ligament reconstruction (n=32)**			
	**Gender**		
		Male	18 (56)
		Female	14 (44)
	**Age in years**		
		Mean age	33
		Age range	21-55

### Mobile App Data

The program was downloaded on to a standard device (mobile phones and tablets) and loaned to the patient for the period of the 30-day pilot. Few technical issues arose during the pilot. In total, there were ten technical inquiries from the 65 patients during the pilot. All of these inquiries related to the patient attempting to access the Internet for personal use on the managed device. All devices and connections worked well throughout the study and were returned on the final follow-up visit.

Patients were asked to log in daily to complete the QoR-9 (data not presented here). The mean number of logins over the 30-day study period was 23.9 (range 7-30) for the breast patients and 19.3 (range 5-30) for the orthopedic patients. The mean number of logins was higher in the first 14 days post-op compared to the 15-30 days post-op for both breast patients (13.4 vs 10.5; *P*<.001) and for the orthopedic patients (13.4 vs 6.0; *P*<.001). The mobile app aggregate response profile data to post surgery quality of recovery indicator questions (QoR 9 modified) is available in Multimedia Appendix 1.

**Figure 2:**
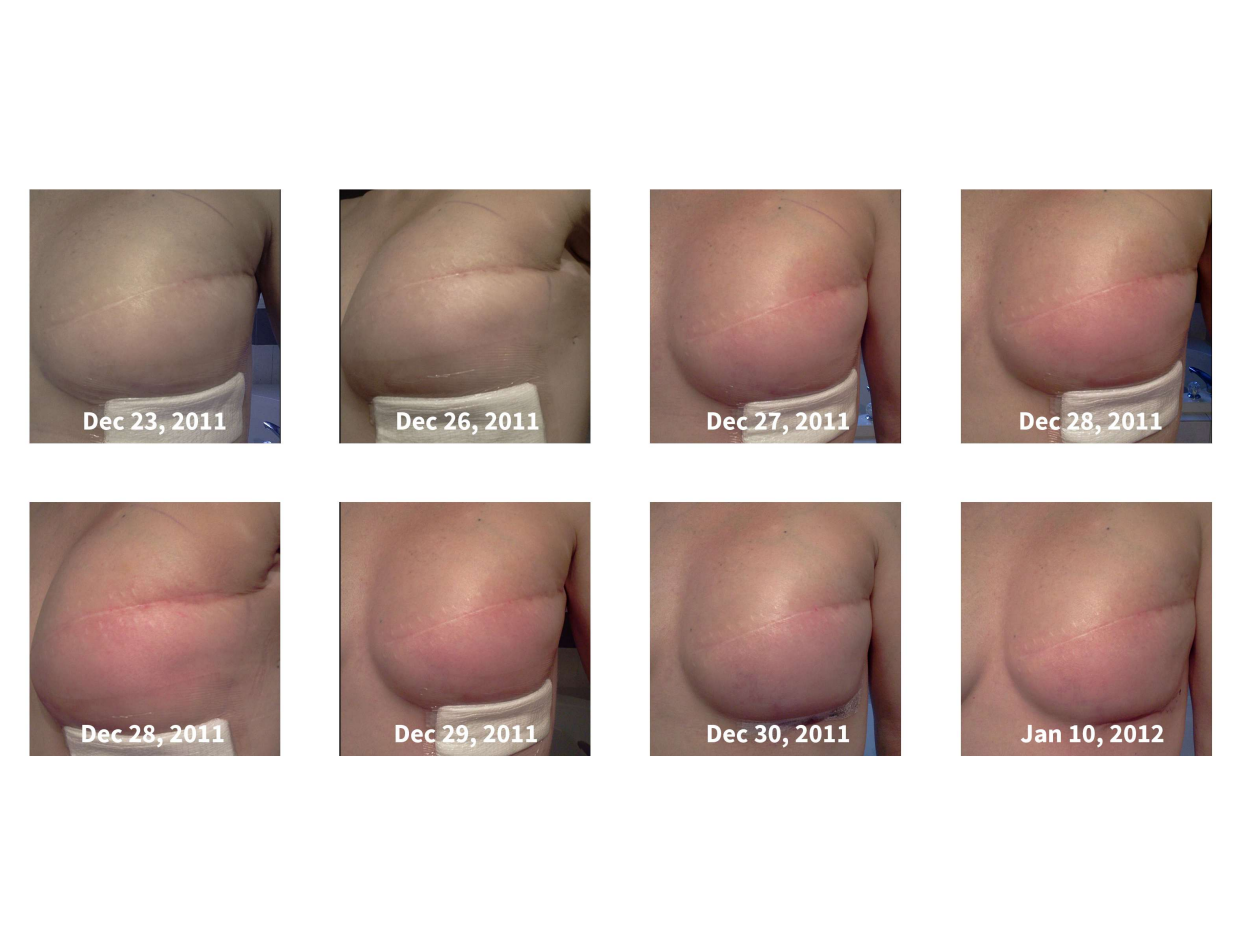
Patient-entered pictures showing left breast of a breast reconstruction patient following insertion of a tissue expander, where the surgeon identified an increasing erythema at 10 days post-op. The sequence of pictures and dates are shown. The patient was placed on antibiotics (over the phone) on Dec. 28th and the erythema starts to recede. The camera quality on most mobile phones is capable of detecting subtle changes in skin tone.

Patients were also asked to upload photographs of the surgical site on a daily basis. Over the 30-day period, 2087 photos were uploaded by the breast patients and 1201 by the orthopedic patients. The mean number of photos uploaded by breast patients was 63 photos (range 11-181) each over the 30-day period, and for orthopedic patients was 38 photos (range 13-160) (*P*=.003). Overall, 82% (range 33%-100%) of the breast patients uploaded at least one photo per day, and 58% (range 30%-100%) of the orthopedic patients uploaded at least one photo per day (*P*<.001). Two potential surgical complications were detected through surgeon viewing of the photographs. Figure 2 shows one of the breast reconstruction patients who was identified with increasing erythema at day 12 post-op. One orthopedic patient was also identified with increased erythema at 5 days post-op. There were no patients who presented clinically with complications that were not identified through monitoring of the mobile phones.

### Post-Recovery Follow-Up Survey for Patients and Post-Recovery Interview

Most of the patients (82%, 53/65) completed the follow-up survey on study completion: 31 breast patients (94%) and 22 orthopedic patients (69%). On a scale from 1 (poor) to 4 (excellent), the mean score for overall satisfaction with using the mobile handheld device was 3.9 for breast patients and 3.7 for orthopedic patients. Most of the patients (87%, 46/53) rated overall satisfaction as excellent, and very few rated it as good (9%, 5/53), fair (4%, 2/53), and poor (0). All patients responded that they would be willing to use the handheld device during a future post-op period.

### Surgeon Follow-up

**Figure 3:**
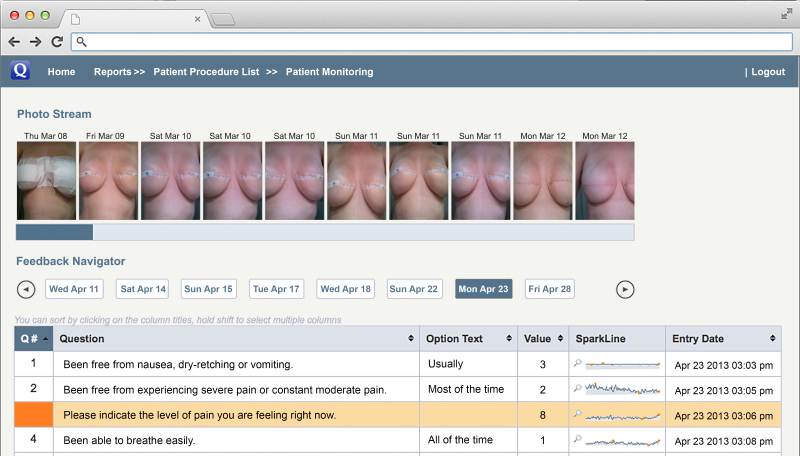
Surgeon's dashboard: The surgeon or care provider can view the patient's quality of recovery all on one screen. The photos show the patient's "selfies" of the surgical site with the date. The QoR-9 questions are listed with the responses. Question #3 is highlighted red because of the abnormally high pain score entered by the patient. The "spark line" represents every score as a point of data on that particular indicator since the start of the monitoring. The pictures can be enlarged by tapping on them.

All three participating surgeons completed the follow-up. Each surgeon followed a mean of 22 patients using the platform. All of the surgeons responded that the platform was user-friendly and intuitive. Specific aspects that enhanced the experience included the easy-to-navigate design, the portability to monitor patients outside of hospital perimeter, and the ability of the technology to improve time efficiency. Surgeons and other care providers reported on the depth of the information that was available in identifying the patient’s recovery trends. New data and insight were apparent in the comparison of the photographic sequence of the surgical site and in the profile of the patient’s recovery. Concerns raised by one surgeon focused on how much additional time would be required by care providers to monitor multiple recovering patients on an ongoing basis. A second point identified the fact that currently (in Canada’s health care system), there is no remuneration code for such activity. Figure 3 depicts an example of the surgeon’s dashboard.

Follow-up visits for patients enrolled in the pilot were scheduled in the usual pattern for each surgeon. When asked whether the surgeons would feel comfortable canceling a follow-up visit if they saw patients were recovering well through the Web portal, all surgeons indicated they would consider canceling the 6-week follow-up for orthopedic surgeries or the first or second follow-up visit for breast-reconstruction surgeries. For canceled follow-up appointments, the orthopedic surgeons indicated that replacing the in-person consultation with a phone call was not an efficient option, and they considered phone calls to be an outdated mode of communication. Rather, surgeons were willing to send an electronic confirmation with personalized feedback to the patients who were progressing well and had their follow-up canceled.

## Discussion

### Principal Findings

Physicians and patients who carry mobile phones are being introduced to the advantage and convenience of “mobile health”, or mHealth. However, there is relatively little research on the feasibility or effectiveness of downloadable apps or software for mobile phones (specifically smartphones) for the remote monitoring of patients following surgery [10].

This study provides evidence that the use of mobile app monitoring with breast reconstruction and orthopedic surgery patients is feasible and acceptable to patients and surgeons.

Patient adherence in using mobile app technology has been demonstrated previously in chronic conditions [16]. However, this pilot study has demonstrated that adherence is also high in acute post-operative patients discharged from hospital within 24 hours after surgery. Patients were asked to log on to the device on a daily basis for 30 days post-operatively. Patient adherence was high; on average, in the first 15 days post-op, patients logged on to the device 13.4 times. This decreased considerably in days 16-30. Logan et al have also reported that adherence also decreases over time in hypertensive patients using mobile devices for blood pressure monitoring [16]. However, with post-operative patients it may be directly related to the type of questions asked of the patients. During the 30 post-operative days, the questions were based primarily on a survey of the patient’s immediate post-op recovery and therefore became less relevant as the weeks passed. A modified questionnaire incorporating different questions relating to features of daily activity (ability of the patient to independently get dressed or return to work) may be more relevant in weeks 3 of a 30-day recovery profile.

Patient satisfaction was very high in this pilot study of breast reconstruction and orthopedic surgery patients. On a scale from 1 (poor) to 4 (excellent), the mean score for overall satisfaction with using the mobile handheld device was 3.9 for breast patients and 3.7 for orthopedic patients. Furthermore, 46 of the 53 patients (87%) rated overall satisfaction as excellent, and all would be willing to use this monitoring in the post-operative period. This patient data supports the acceptance of this type of mobile monitoring. These findings are consistent with other studies evaluating the acceptance of the use of mobile technology in other medical conditions including hypertension, congestive heart failure, and diabetes [16-19]. Generally, it has been reported that patients have an overall positive attitude towards mobile technology [20].

In addition to feasibility of patients using the device, we have also demonstrated the feasibility of surgeons monitoring post-operative patients. Through the platform, the surgeons had a first-time view of daily patient recovery between discharge from hospital and the first follow-up visit through the use of the quality of recovery data and photographs. Observing the incisions in sequence allowed for a new type of indicator assessment for surgeons and an opportunity for intervening in the early development of post-operative infections. The resolution and quality of cameras available on most mobile phones are now capable of detecting subtle color changes in skin tone. As a result, complications were observed in real-time and allowed for the identification of complications prior to scheduled follow-up visits. Furthermore, there were no complications identified at follow-up visits that had not been identified using the mobile phone monitoring. The use of photographic imaging using mobile phones in post-operative monitoring of microvascular free tissue transplantations has previously been examined in post-operative patients; Engel et al [21] used a prospective study to compare the accuracy rate in detecting complications using mobile phone photographic assessments compared to in-person examinations. They reported that the remote mobile phone photography assessment had a comparable accuracy rate, and importantly, had a shorter response time compared with an in-person visit. In the post-operative period, it is critical that complications be identified promptly and treated.

Our research and that of others, demonstrates that mobile phone monitoring using photographs for post-operative patients is feasible for patients and effective at detecting complications for surgeons. Consequently, in our study, surgeons identified that with the mobile monitoring they would feel comfortable reducing post-operative follow-up visits if patients were using mobile monitoring. Other modalities of telemedicine, including Skype, have previously been shown to decrease the number of unscheduled post-operative visits in patients with total joint arthroplasty [22]. The reduction in the number of post-operative visits has financial implications. We have previously reported that mobile app follow-up care is cost-effective from a societal and health care system perspective [23].

The most common issues raised in regards to using mobile apps in health care are privacy and data security, funding, a lack of good examples of the efficacy and cost effectiveness in practice, and the need for more high-quality research [1]. With appropriate attention to privacy and security, these concerns can be addressed in a comprehensive manner. Given the fact that this may be considered “new data”, one must be aware of who owns the data and where the data should go in regards to patient care. In this study, we assumed the data to belong to the patient and a copy of the results, in accordance with the Review of Ethics Board for the Women’s College Hospital Research Institute, were sent to the patient’s hospital chart.

### Limitations

There are limitations to the current study. Information regarding the patient’s race, ethnicity, or socioeconomic status was not collected. The study sample was relatively young and as a result may have been more comfortable with technology. In addition, the sample of surgeons was small. Further large-scale studies are required to ensure generalizability of the results. Furthermore, it will be important to evaluate the cost-effectiveness of using this type of technology, and further studies are planned to elucidate the cost and efficiency details with greater clarity.

### Conclusions

This study sets the stage for future studies of this nature in different patient populations, both acute and chronic. This type of at-home monitoring using mobile technology appears to be feasible and acceptable for breast reconstruction and orthopedic surgery patients. Future studies can leverage this initial proof of concept and explore in more detail the use of patient-reported recovery information and mobile technology. The results of this pilot study provide a possible solution that supports the current shift in health care from inpatient care to ambulatory care and increased emphasis on recovery in the home.

## Multimedia Appendix 1

Mobile App Aggregate Response Profile data to Post Surgery Quality of Recovery Indicator questions (QoR 9 modified). Two post surgical populations Breast Reconstruction and Orthopedic (ACL repair).

## Abbreviations

AES: advanced encryption standard
HL7: Health Level Seven International
IaaS: infrastructure as a service
ITIL: Information Technology Infrastructure Library
QoR-9: Quality of Recovery questionnaire
SaaS: cloud computing services
SAS70: Statement on Auditing Standards No. 70
SIM card: international mobile subscriber identity
TLS/SSL: transmission transport layer security/secure sockets layer
VAS Scale: Visual Analogue Scale
