# Supplementary material for: Using a Mobile App for Monitoring Post-Operative Quality of Recovery of Patients at Home: A Feasibility Study
Source: JMIR Mhealth Uhealth. 2015 Feb 12;3(1):e18. doi: 10.2196/mhealth.3929 (PMC4342621; doi:10.2196/mhealth.3929)
Supplement: Supplementary file 1 [file mhealth_v3i1e18_app1.pdf]

Mobile App Aggregate Response Profile data to Post Surgery Quality of Recovery Indicator questions (QoR 9 modified).

Two post surgical populations Breast Reconstruction and Orthopedic (ACL repair).

Pain

The data from the mobile application shows that the level of pain post-surgery in the breast reconstruction group gradually declined over a 30 day period, with patients on average reporting decreased pain by day four post-surgery. A slightly more sporadic pattern of pain is seen in the orthopedic ACL group; however a declining trend can be seen over the 30 days following surgery.

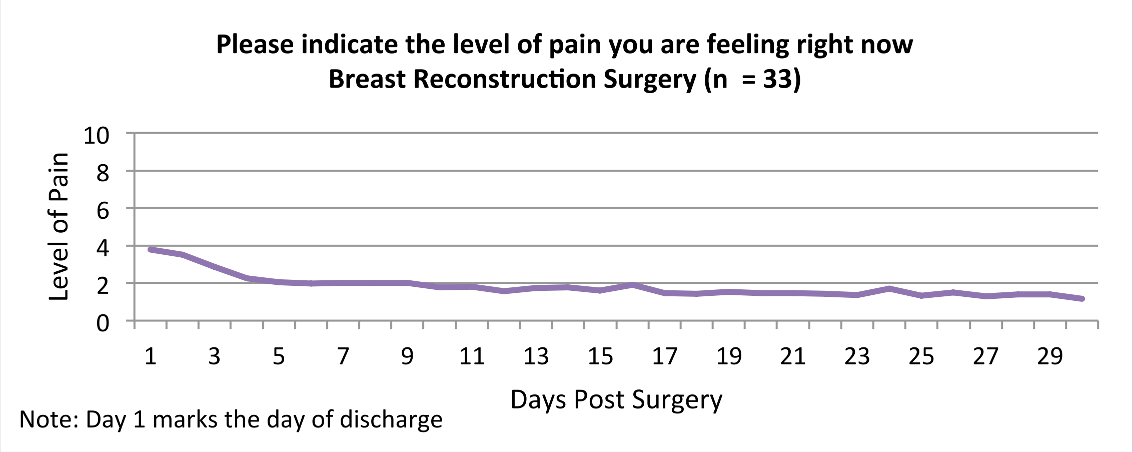

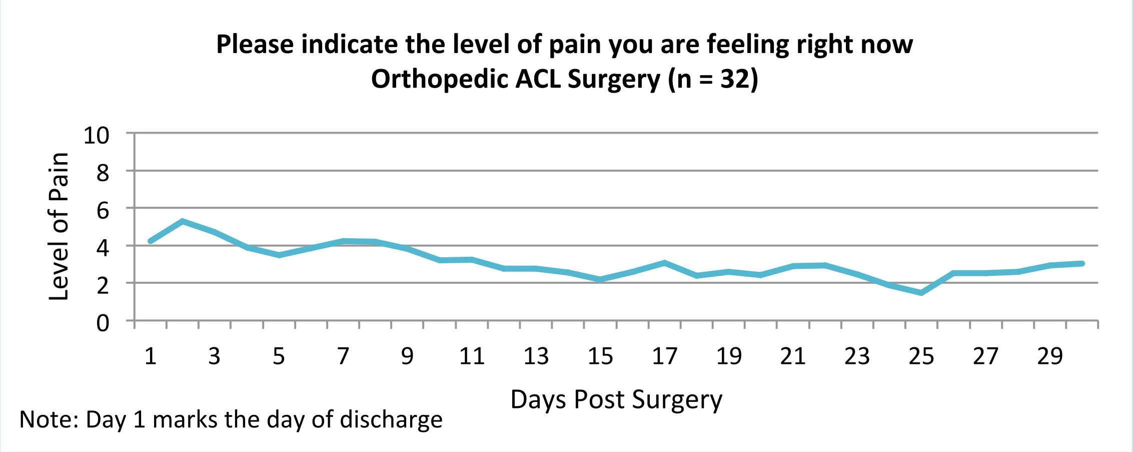

Anxiety

The level of anxiety and nervousness remained fairly low among both groups throughout the recovery period.

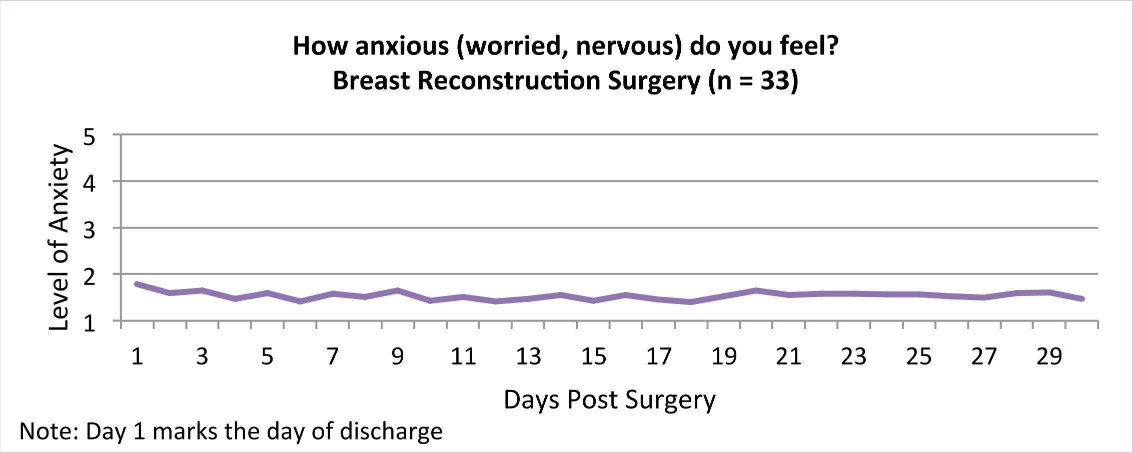

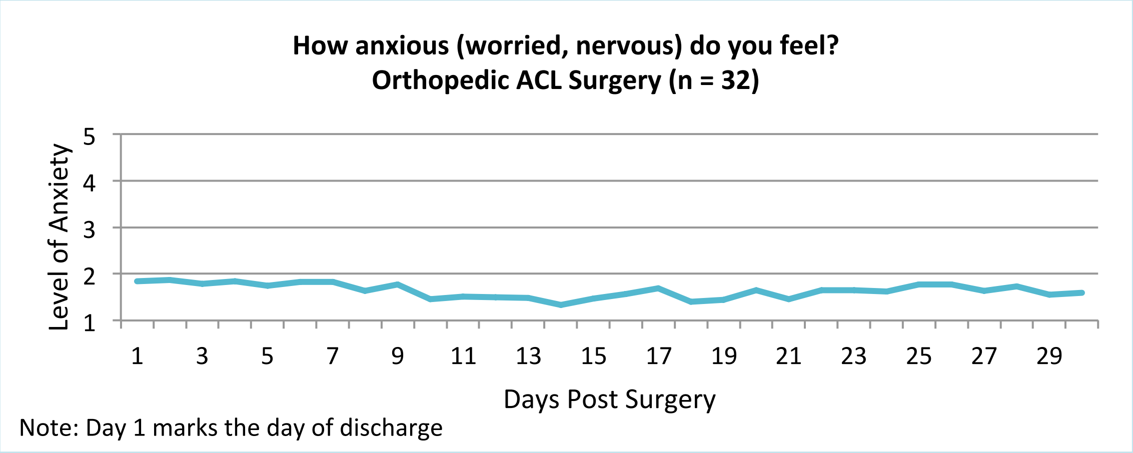

General Well being

The level of discomfort experienced by breast reconstruction patients gradually decreased over 30 days following surgery. A slight spike in discomfort was witnessed between day of discharge (day 1) and the second day of being home. This is perhaps due to patients adjusting to the home environment (i.e. the addition of stairs, walking, etc.). In the orthopedic ACL patient group, the decline in discomfort occurred more gradually and patients had a higher level of pain throughout recovery.

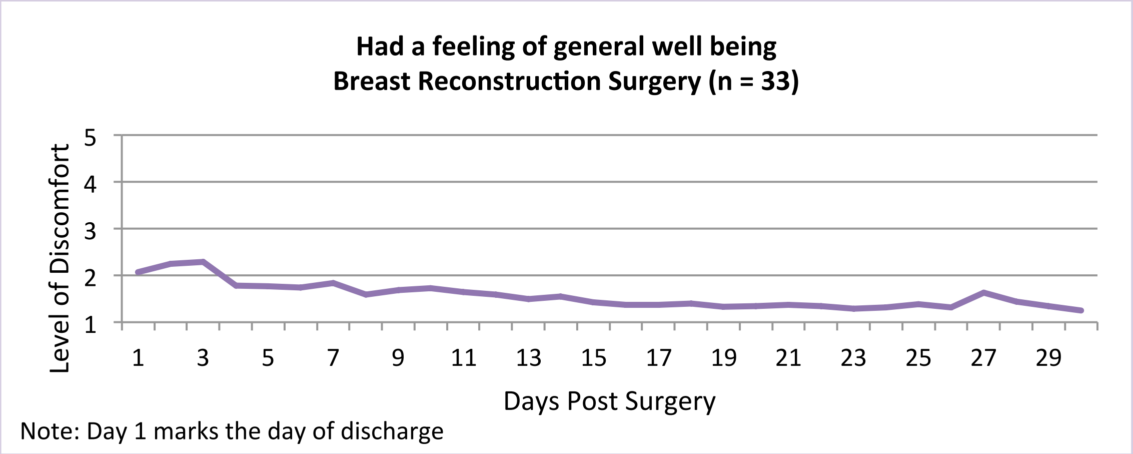

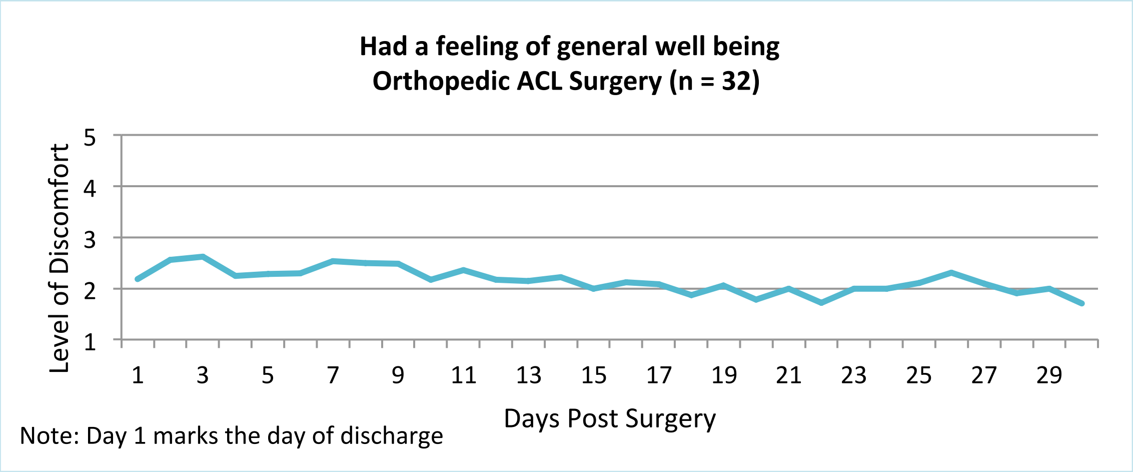

Support from Others

All patients had higher levels of support during the first few days post-surgery. This is to be expected immediately post-surgery with the need for support dropping as recovery progresses.

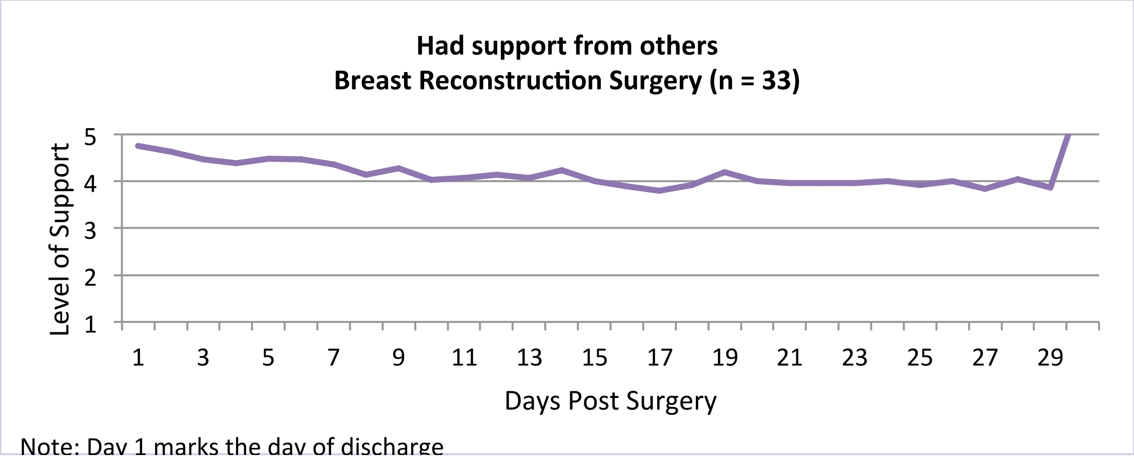

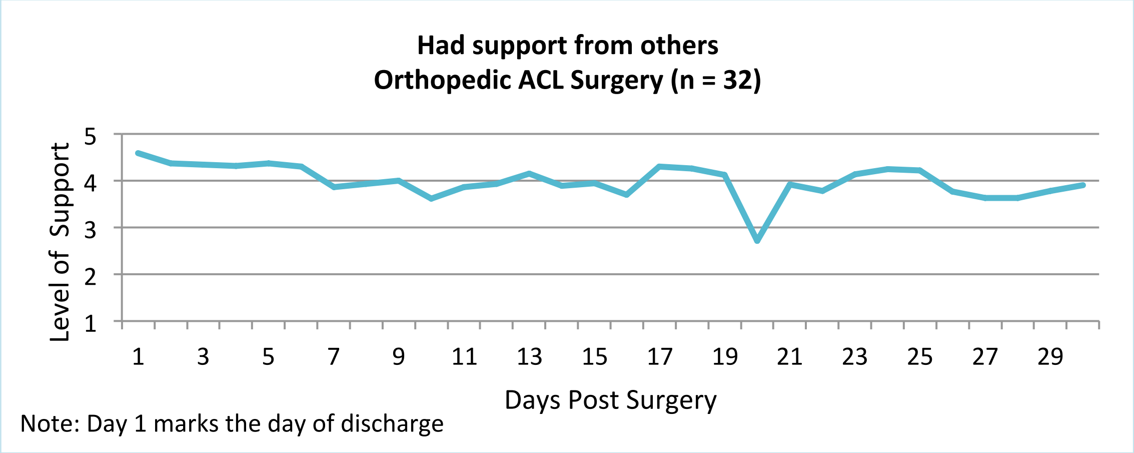

Free from Pain

Looking at trends over time, severe pain or constant moderate pain in the breast reconstruction group was primarily high up to four days following surgery. A more sporadic trend of pain was experienced among the orthopedic patients and could reflect increased pain as physical therapy begins.

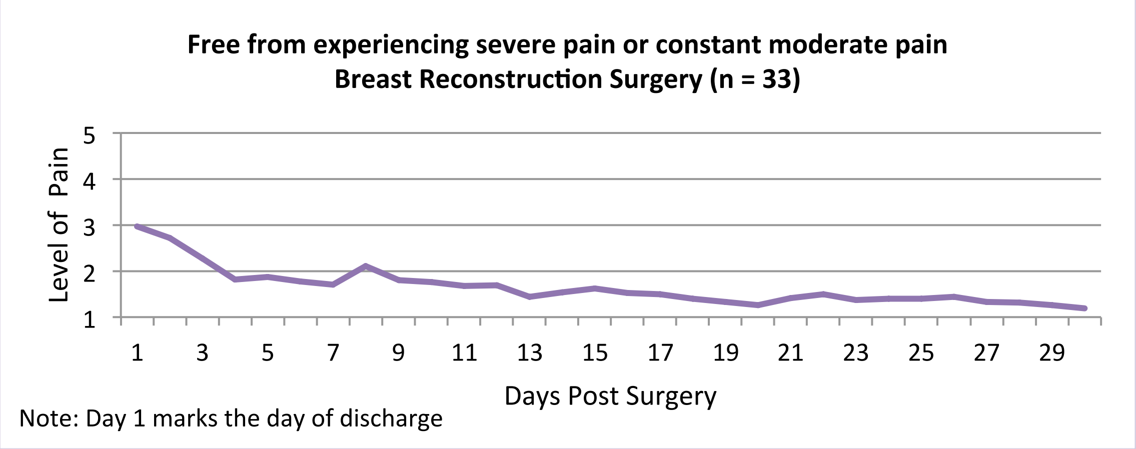

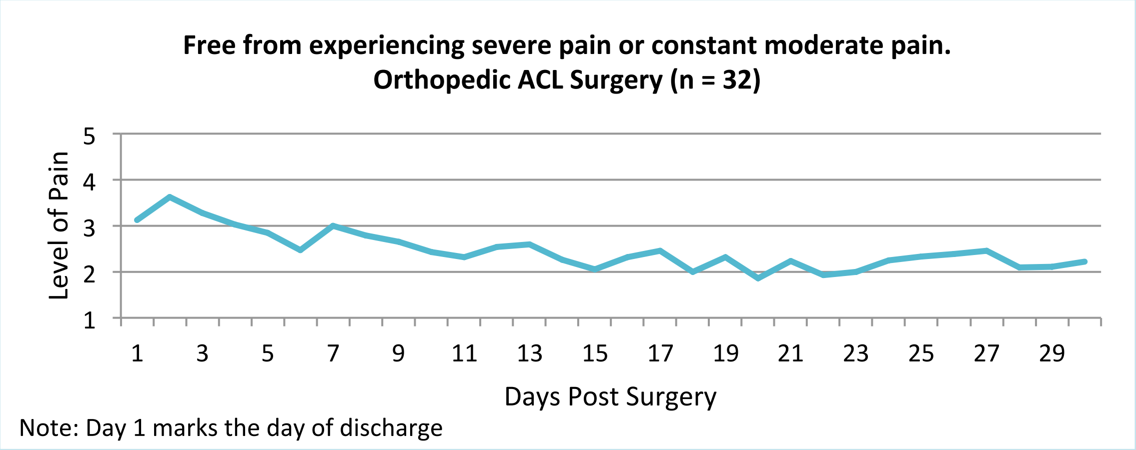

Breathing

Among both groups, patients indicated being able to breathe easily almost all the time to most of the time. It appears that orthopedic ACL patients had a slightly higher difficulty in breathing throughout the recovery period.

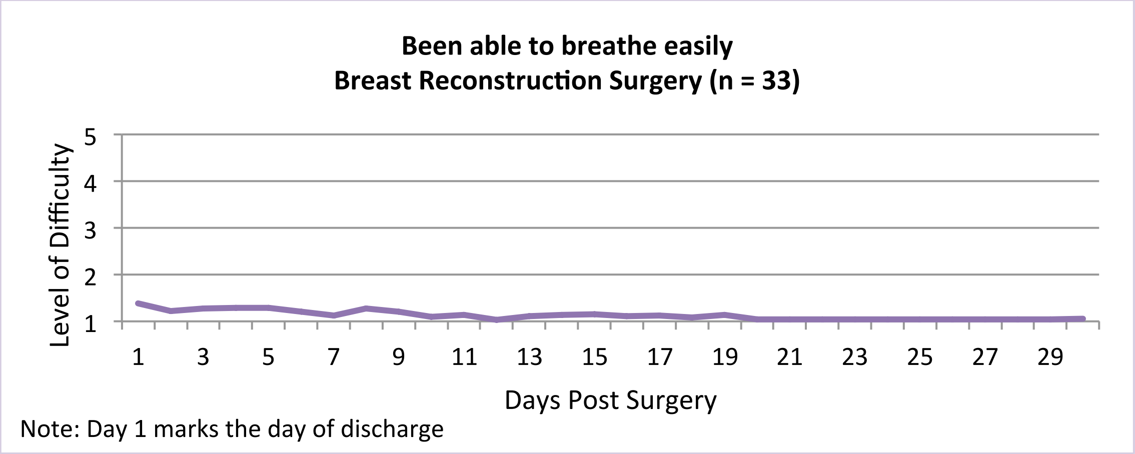

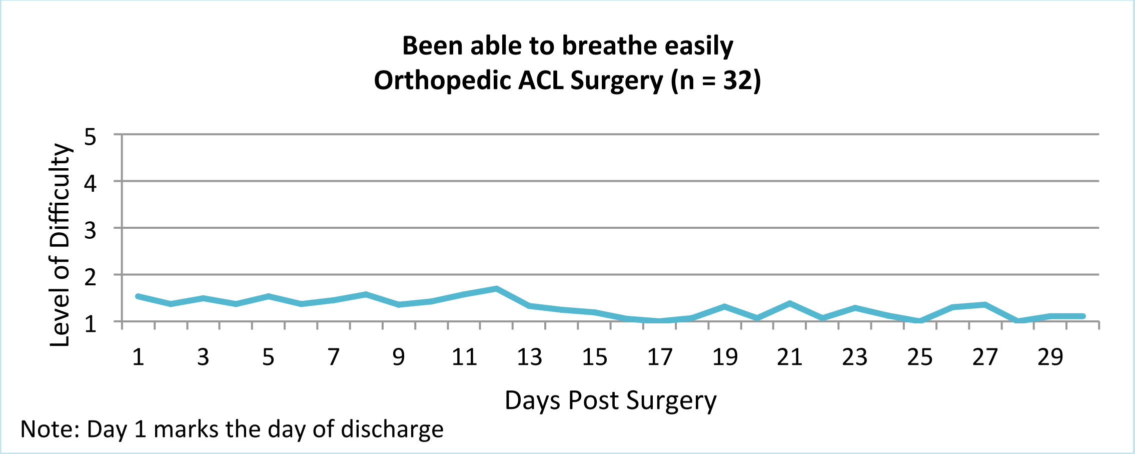

Unaided Personal Hygiene

From the application data we see that breast reconstruction patients experienced initial difficulty in their ability to look after personal toilet and hygiene unaided. Difficulty dropped substantially between day 1 and day 2 post-discharge. Orthopedic patients required assistance with personal hygiene for a longer period of time in comparison to the breast reconstruction group (Figure 42). This is likely due to the nature of orthopedic ACL surgery which can cause mobility difficulties.

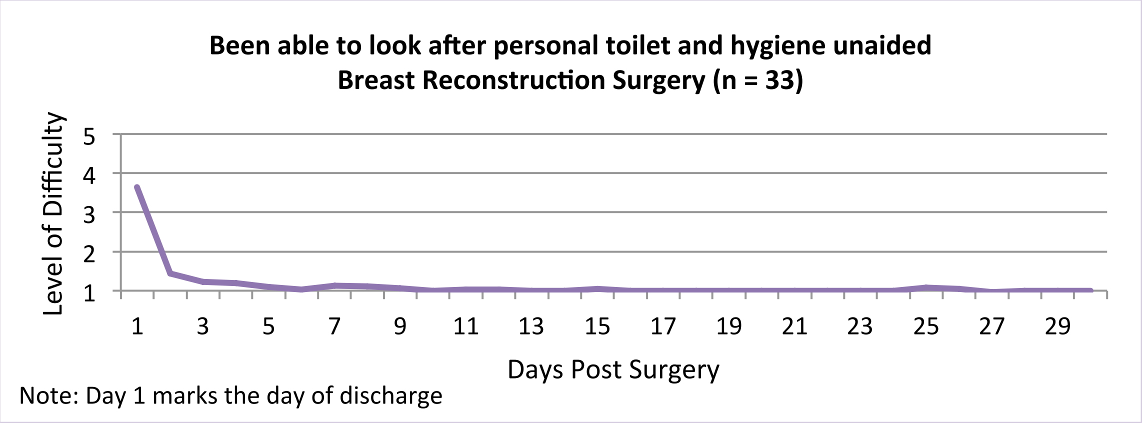

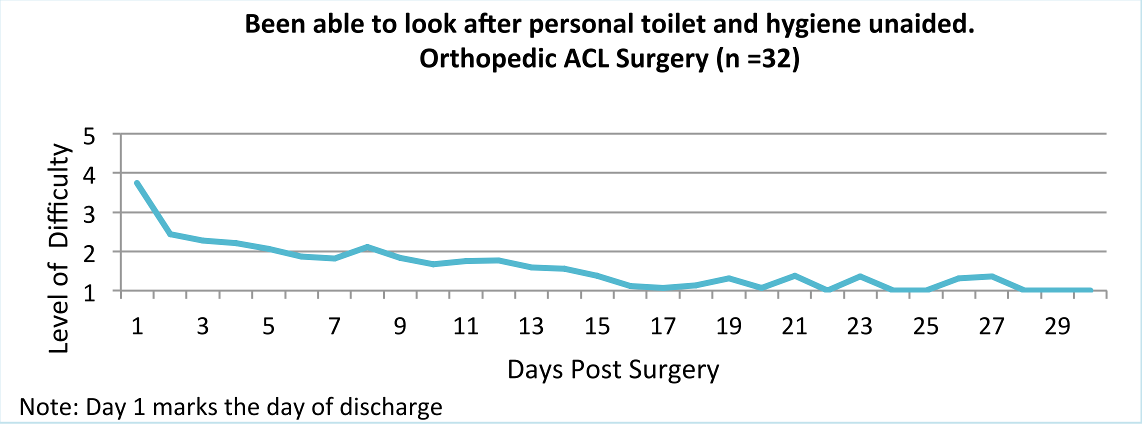

Able to Pass Urine

Looking at trends in recovery, patients reported being able to pass urine without difficulty fairly quickly following surgery. However, it appears that it took a longer amount of time for the orthopedic patients to return to normal urine function in comparison with breast reconstruction patients.

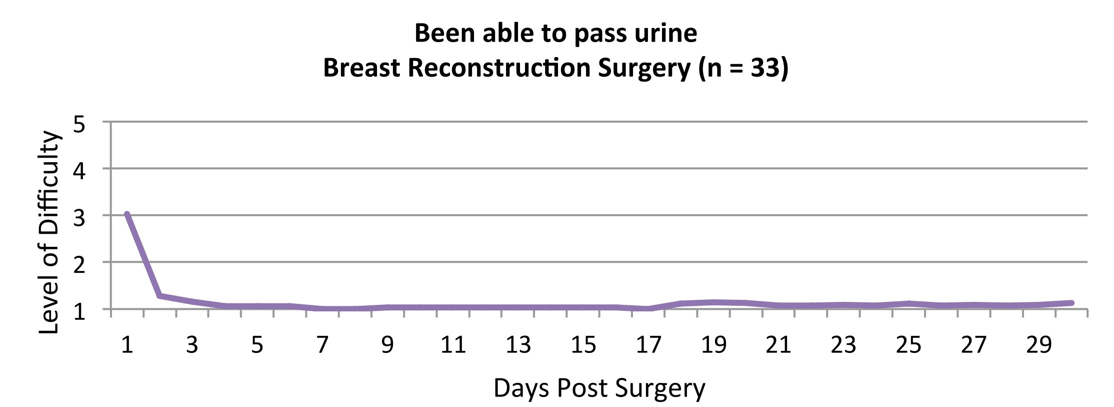

Note: Day 1 marks the day of discharge

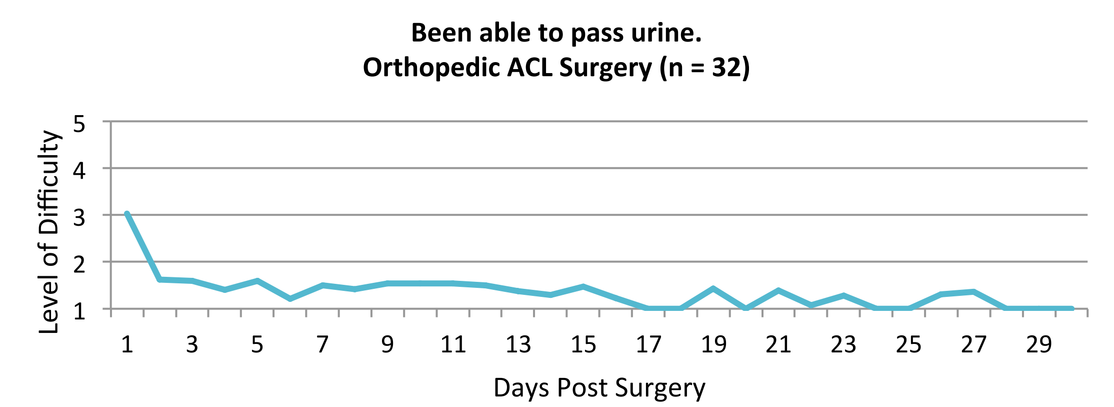

Note: Day 1 marks the day of discharge

Normal Bowel Function

For both groups, bowel function returned to normal within 10 days of surgery and remained fairly steady thereafter. The orthopedic ACL group exhibited a slower return to normal bowel function than the breast reconstruction group.

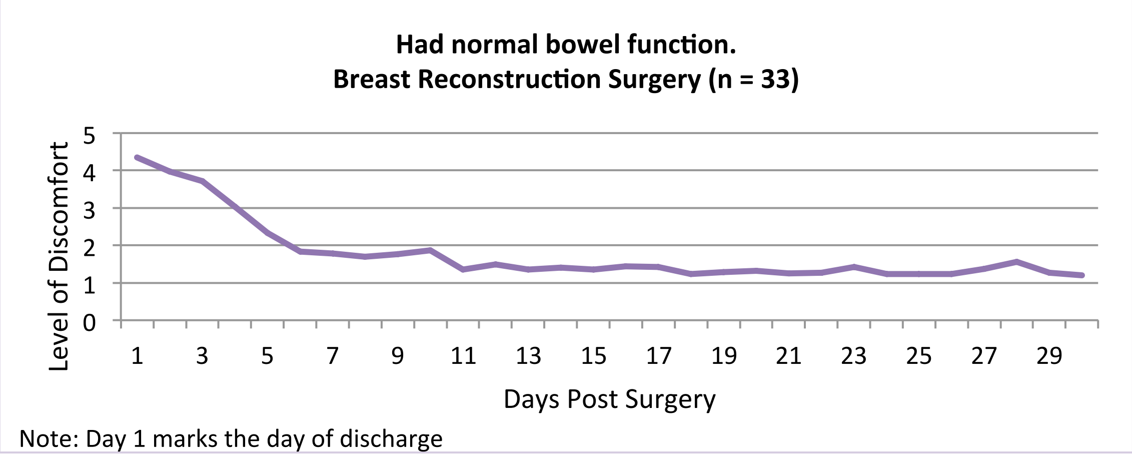

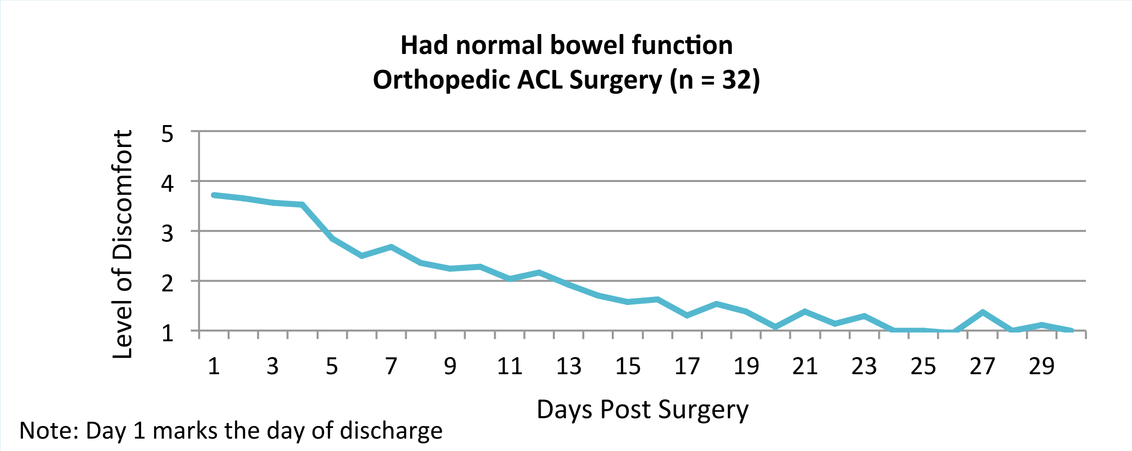

Free from Nausea, Dry-retching and Vomiting

Looking at Figures, breast reconstruction patients experienced an initial higher level of discomfort from nausea, dry-retching or vomiting compared to orthopedic ACL patients. However, the discomfort in both groups fades away roughly two weeks following surgery. The discomfort in breast reconstruction patients has a sharper and more rapid decline post-surgery in comparison to the orthopedic ACL patients.

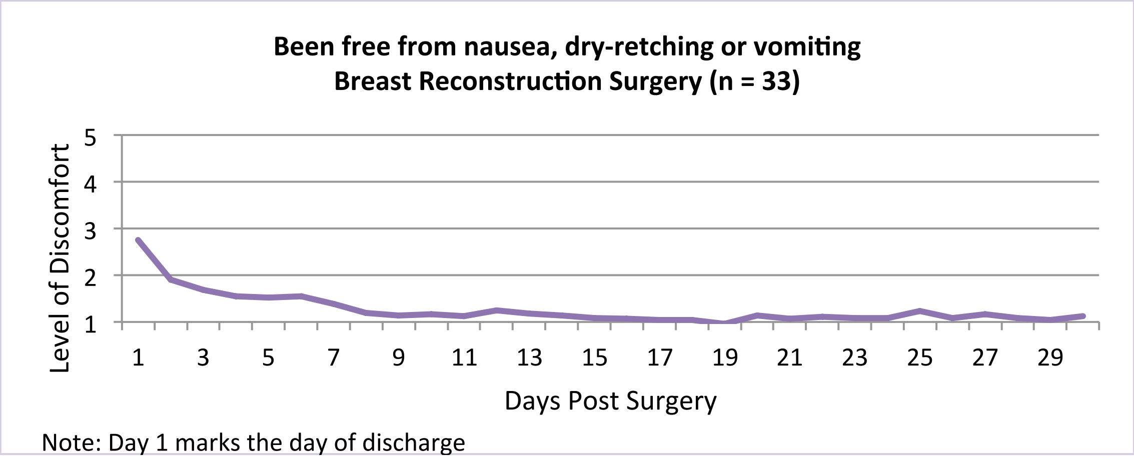

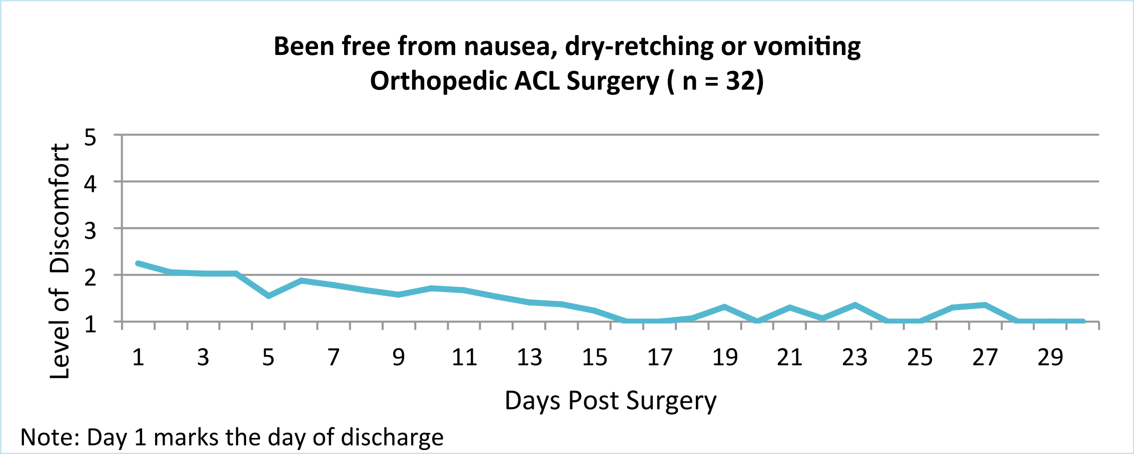

Comprehend Instructions

Little difference in the level of difficulty in comprehending instructions was illustrated between days immediately following surgery and 30 days later in the breast reconstruction group. The orthopedic ACL group showed slightly more difficulty in understanding instructions and advice especially in the first two weeks post-discharge.

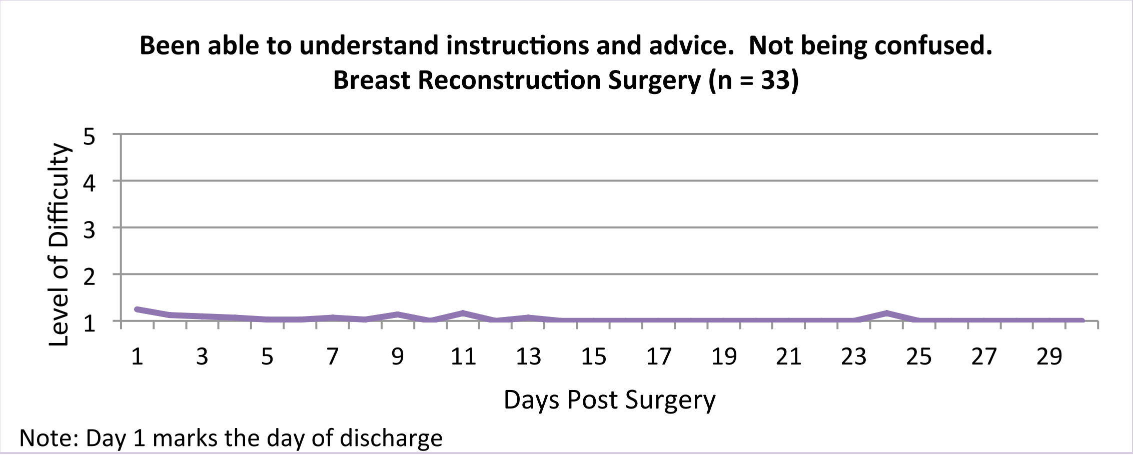

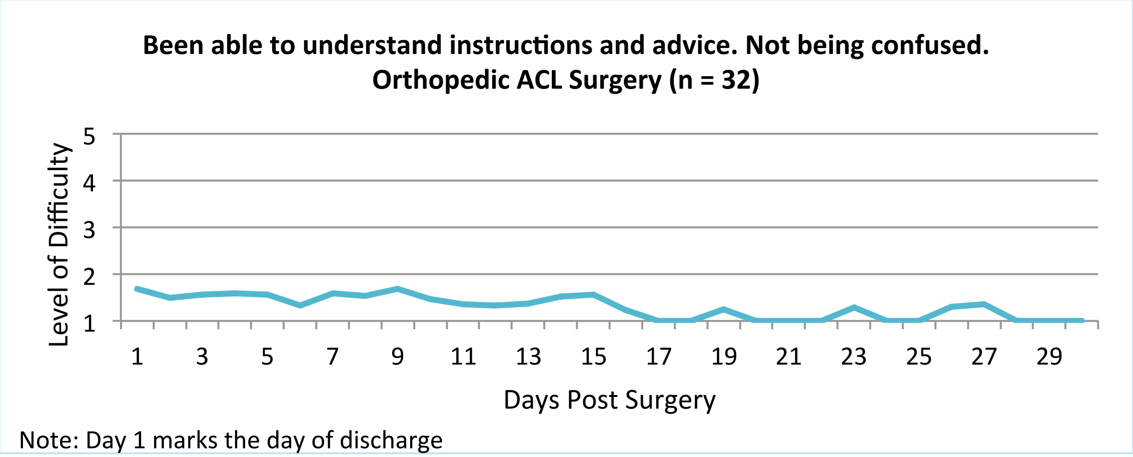

Free from headache, backache or muscle pains

Both groups of patients reported low levels of aches during the 30 day recovery period with a slow decrease in discomfort over time.

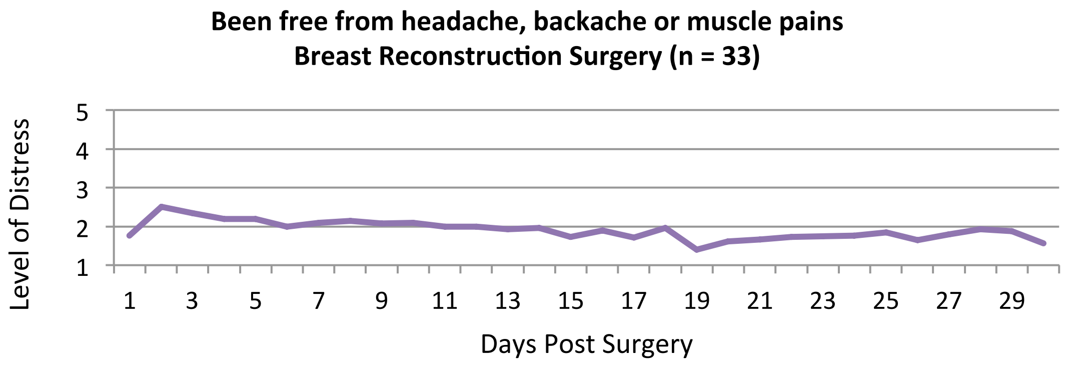

Note: Day 1 marks the day of discharge

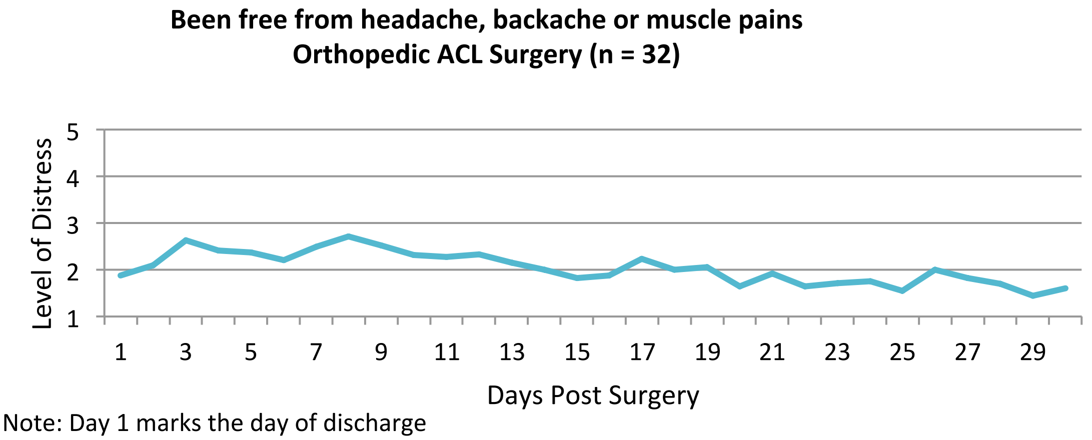

Note: Day 1 marks the day of discharge

Fluid Drained from Wound (abdominal drains) of Breast Reconstruction Patients

The following figures demonstrate the trend of abdominal fluid drainage for one patient with breast reconstruction surgery requiring an abdominal drain (TRAM - *transverse rectus abdominus myocutaneous* surgery). In the pilot there was only one patient with this type of surgery and drain and the graph is displayed to show the type of results physicians can see when patients use the mobile application for reporting.

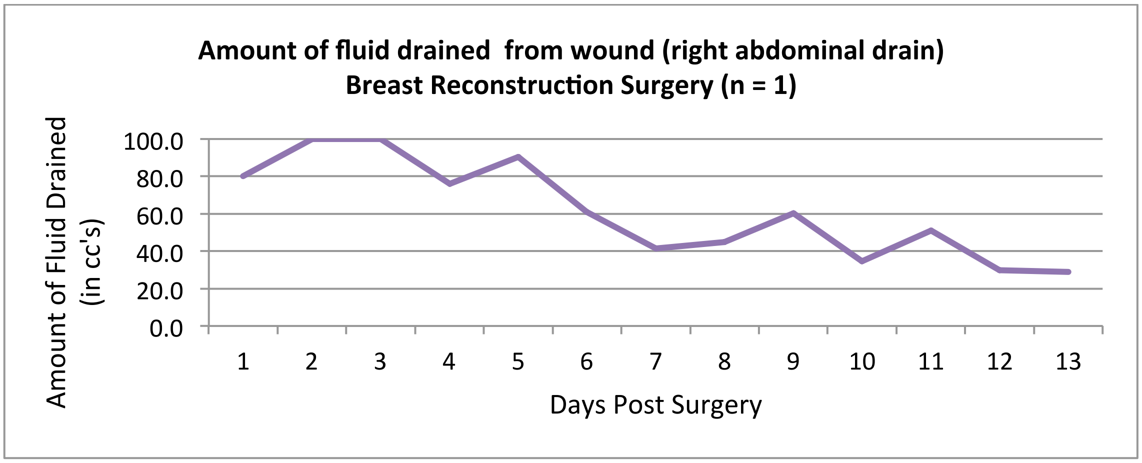

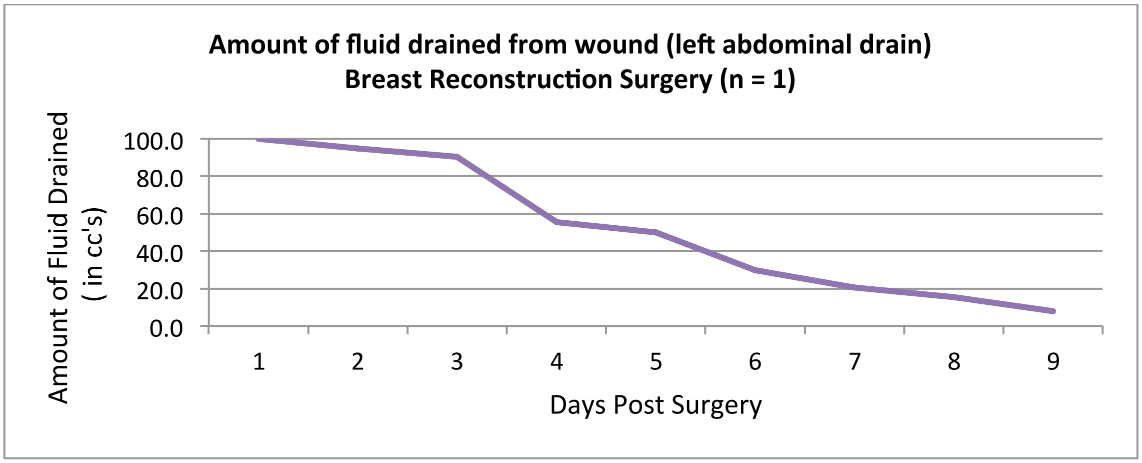

Amount of Fluid drained from Wound (Breast Drains)

Again, these indicators of drainage from the surgical site only apply to the breast reconstruction patients in the pilot. The following figures show the amount of fluid drained from right and left breasts.

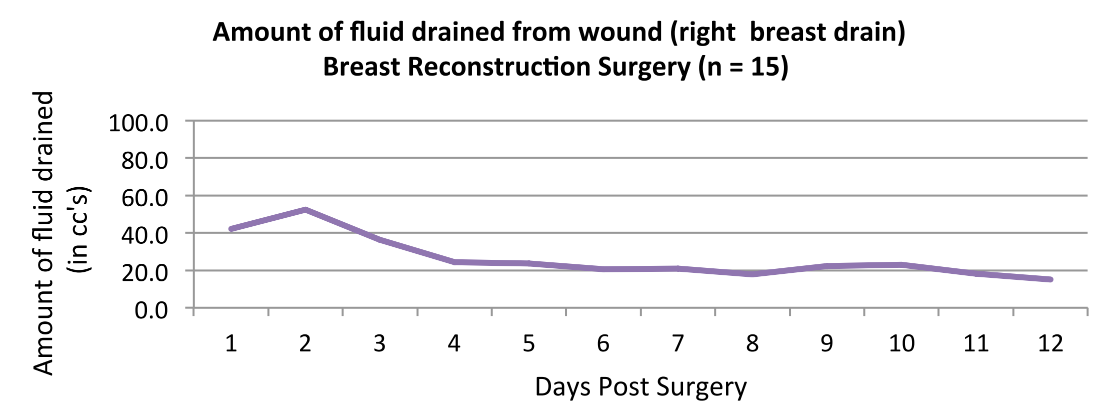

Note: Day 1 marks the day of discharge

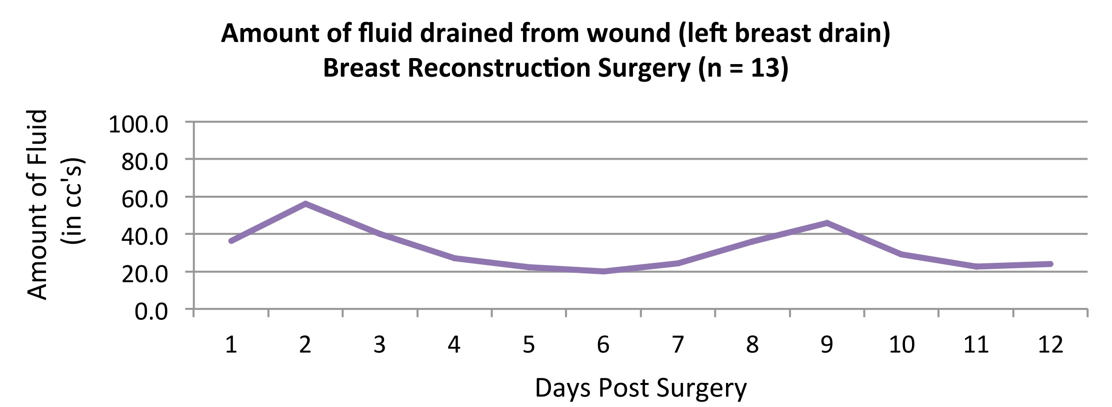

Note: Day 1 marks the day of discharge

Difficulty with Mobility among Orthopedic ACL Patients

The following figures illustrate indicators that describe the level of difficulty in mobility that orthopedic ACL patients experience. These indicators include difficulty in performing activities of daily living, climbing up and down the stairs, walking, and standing. As expected with normal recovery from orthopedic ACL surgery, a gradual decrease in the level of difficulty in mobility is demonstrated in these graphs.

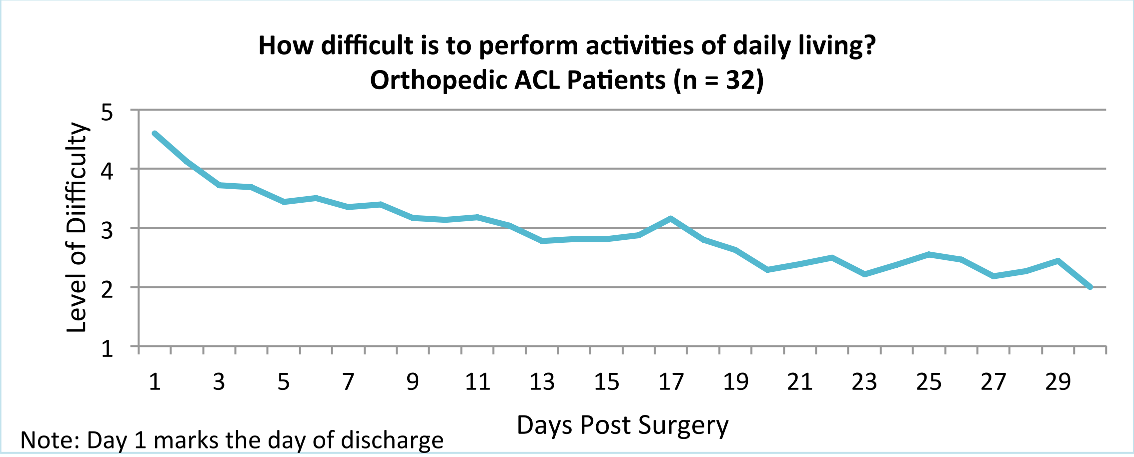

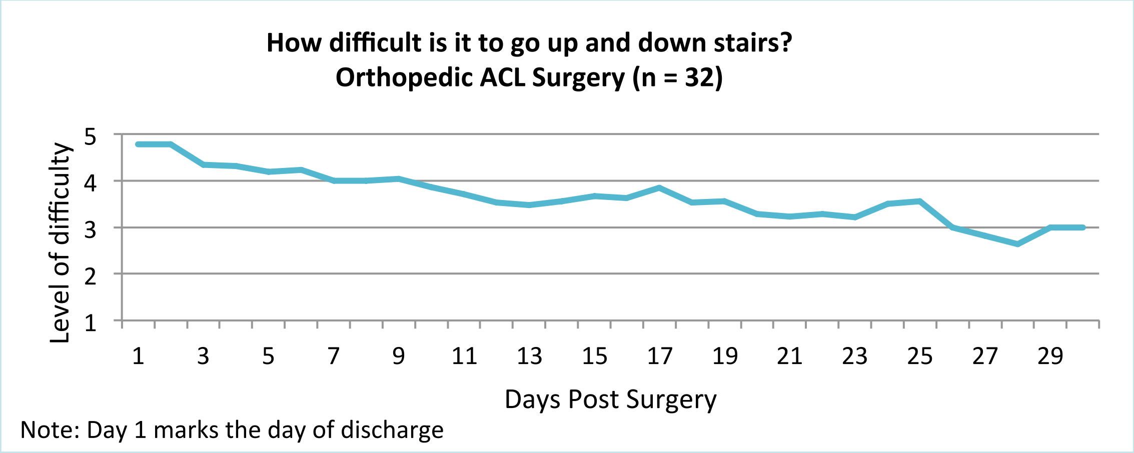

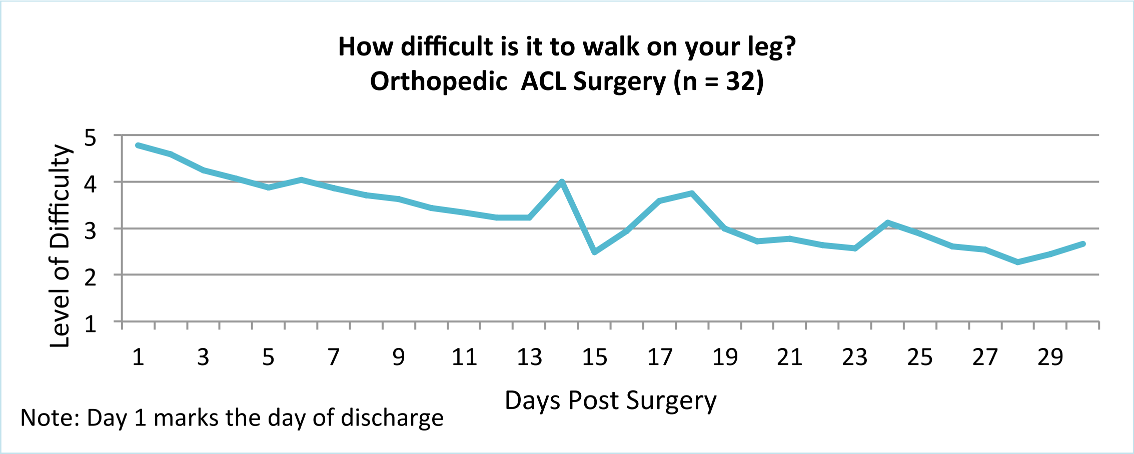
How difficult is it to walk on your leg?
Orthopedic ACL Surgery (n = 32)
Level of Difficulty
5
4
3
2
1
1
3
5
7
9
11
13
15
17
19
21
23
25
27
29
Days Post Surgery
Note: Day 1 marks the day of discharge

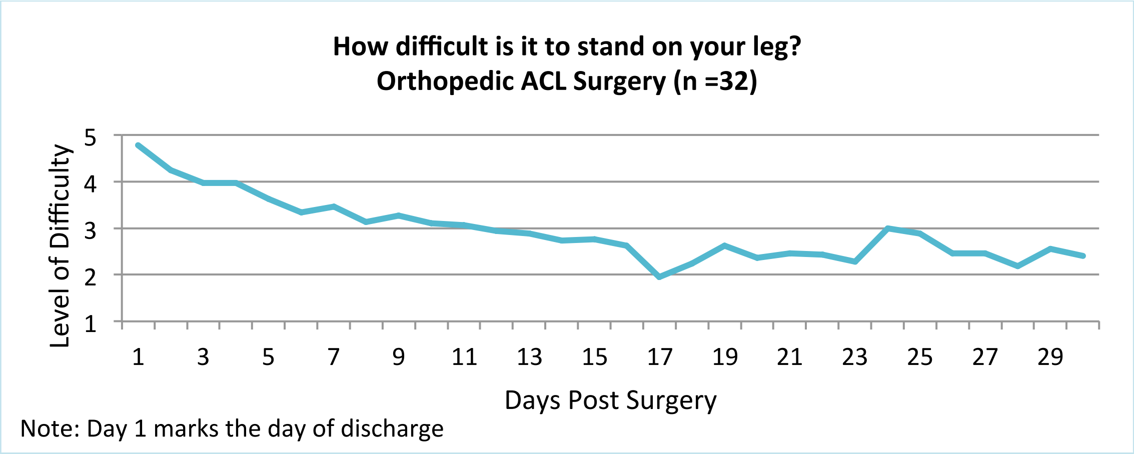
How difficult is it to stand on your leg?
Orthopedic ACL Surgery (n =32)
Level of Difficulty
5
4
3
2
1
1
3
5
7
9
11
13
15
17
19
21
23
25
27
29
Days Post Surgery
Note: Day 1 marks the day of discharge
